# Current Progress and Future Perspectives in Contact and Releasing-Type Antimicrobial Coatings of Orthopaedic Implants: A Systematic Review Analysis Emanated from In Vitro and In Vivo Models

**DOI:** 10.3390/idr16020025

**Published:** 2024-03-26

**Authors:** Angelos Kaspiris, Elias Vasiliadis, Evangelia Pantazaka, Ioanna Lianou, Dimitra Melissaridou, Matthaios Savvidis, Fotios Panagopoulos, Georgios Tsalimas, Michail Vavourakis, Ioannis Kolovos, Olga D. Savvidou, Spiros G. Pneumaticos

**Affiliations:** 1 Third Department of Orthopaedic Surgery, School of Medicine, National and Kapodistrian University of Athens, “KAT” General Hospital, Nikis 2, 14561 Athens, Greece; eliasvasiliadis@yahoo.gr (E.V.); georgetsalimas@yahoo.com (G.T.); michail.vavourakis@outlook.com (M.V.); kolovioan@gmail.com (I.K.); spirospneumaticos@gmail.com (S.G.P.); 2 Synthetic Organic Chemistry Laboratory, Department of Chemistry, University of Patras, 26504 Patras, Greece; evapantazaka@upatras.gr; 3 Department of Orthopedic Surgery, “Rion” University Hospital and Medical School, School of Health Sciences, University of Patras, 26504 Patras, Greece; jolianou@hotmail.com (I.L.); panfo97@gmail.com (F.P.); 4 First Department of Orthopaedic Surgery, School of Medicine, National and Kapodistrian University of Athens, “ATTIKON” University Hospital, Rimini 1, 12462 Athens, Greece; dimitramelissaridi@gmail.com (D.M.); olgasavvidou@gmail.com (O.D.S.); 5 Second Orthopedic Department, 424 General Military Hospital, 56429 Thessaloniki, Greece; makisorto@hotmail.com

**Keywords:** preclinical studies, orthopedic implants, antimicrobial coatings, biocompatibility, osseointegration

## Abstract

*Background:* Despite the expanding use of orthopedic devices and the application of strict pre- and postoperative protocols, the elimination of postoperative implant-related infections remains a challenge. *Objectives:* To identify and assess the in vitro and in vivo properties of antimicrobial-, silver- and iodine-based implants, as well as to present novel approaches to surface modifications of orthopedic implants. *Methods:* A systematic computer-based review on the development of these implants, on PubMed and Web of Science databases, was carried out according to the Preferred Reporting Items for Systematic Reviews and Meta-Analyses guidelines. *Results:* Overall, 31 in vitro and 40 in vivo entries were evaluated. Regarding the in vitro studies, antimicrobial-based coatings were assessed in 12 entries, silver-based coatings in 10, iodine-based in 1, and novel-applied coating technologies in 8 entries. Regarding the in vivo studies, antimicrobial coatings were evaluated in 23 entries, silver-coated implants in 12, and iodine-coated in 1 entry, respectively. The application of novel coatings was studied in the rest of the cases (4). Antimicrobial efficacy was examined using different bacterial strains, and osseointegration ability and biocompatibility were examined in eukaryotic cells and different animal models, including rats, rabbits, and sheep. *Conclusions:* Assessment of both in vivo and in vitro studies revealed a wide antimicrobial spectrum of the coated implants, related to reduced bacterial growth, inhibition of biofilm formation, and unaffected or enhanced osseointegration, emphasizing the importance of the application of surface modification techniques as an alternative for the treatment of orthopedic implant infections in the clinical settings.

## 1. Introduction

Postoperative implant-related infection, following bone defect and primary or revision total joint arthroplasties, is a reality and remains a challenge in orthopedics with devastating clinical consequences despite the application of strict protocols of aseptic techniques and perioperative antibiotics [1,2]. As surgery techniques and orthopedic implants are constantly being optimized, so is the need for these implants by patients, and hence, so is the possibility of infection occurrence. Implant devices are, therefore, not a panacea; their use is not devoid of issues and their introduction bears the risk of them being colonized by bacteria, which will ultimately lead to the development of implant-related infection [3]. Implant removal for the elimination of infection not only impacts patients’ health and quality of life but also poses a huge financial burden, which relates to the repetition of surgical procedures, long-term hospitalization, and medication costs, visits to physicians, as well as time off work [2,4,5,6]. Implant-related infection is immensely difficult to avoid or treat; it may impede the healing process and result in implant failure, chronic osteomyelitis, sepsis, and even death [4,7].

For the most part, difficulty in the treatment of infection rests on several factors, such as bacteria affinity for the implants’ surface, adhesion, and biofilm formation, which is a crucial step, and the development of antimicrobial resistance [1]. The process of biofilm formation, which helps many bacterial species to adapt to various stresses, comprises cellular attachment (reversible and irreversible) to surfaces, microcolony formation, maturation, and dispersion of single cells from the biofilm. Biofilm formation decreases sensitivity to host immune defenses, circumvents systemic antimicrobial regimens, and increases resistance to antimicrobials [5,8,9]. *Staphylococcus aureus*, *Staphylococcus epidermidis*, *Pseudomonas aeruginosa*, as well as methicillin-resistant *S. aureus* (*MRSA*) are notorious for the formation of biofilms, which are implicated in implant failure.

Systemic application of antimicrobials, as the first-line treatment strategy, is associated with poor site accessibility and increased toxicity [7]. The preclinical use of antimicrobials for the prophylaxis of dreaded implant-associated infections has been reported for a long time [1,5,7,10,11]. A plethora of surface modifications by coating the implants’ surfaces with appropriate molecules have been developed, and a subset will be reviewed here.

The antimicrobial activity of these implants is mainly based on drug-release or non-release methods. Non-release methods refer to materials that can defend the adhesion of microbes and avoid access to the coated material and biofilm formation [12]. To this end, the ideal coating would need to eradicate bacterial growth, inhibit adhesion and biofilm formation, and then facilitate bone formation. It would, therefore, have to achieve a balance between cytotoxicity and antimicrobial efficacy and hence support the adhesion of bone-related cells (e.g., osteoblasts) while inhibiting bacterial adhesion. In the case of Intraosseous Amputation Prosthesis, achievement of tissue integration requires eukaryotic rather than bacteria cells to win the “race for the surface”, keeping in mind that bacteria may as well reside in the surrounding tissue, a bit further away from the implant surface [11,13]. Another important aspect of the delivery system is release kinetics, both in vitro and in vivo. These aspects are reflected in the in vitro and in vivo assessments of the reported categories of implants in this review.

Development and/or identification of biomaterials that combine both antimicrobial and osteogenesis activities are promising approaches for infected bone repair, with a focus on the interface of the implant and the surrounding tissue. Notwithstanding the non-specific effects, poor release kinetics, and toxicity profiles, a lot of effort is now concentrated on modifying the implants’ properties, rendering them less susceptible to infections [1,7]. Amongst the coatings developed during the last years, antimicrobial-coated and silver-coated biomaterials have been extensively studied [2,10,14]. In addition, iodine and a variety of other coatings (including metal-, vitamin-E (VE), antimicrobial peptides (AMPep)) have gained attention. Coatings can be classified as active or passive [10], based on whether they allow or not release of the antimicrobial agents, with the majority of the ones reviewed here being passive.

Although clinical translation has been relatively limited, there are antimicrobial implant coatings available in clinics nowadays [15], and they have so far shown promise and fewer drawbacks. Nonetheless, there is a pressing need for more knowledge regarding the in vitro and in vivo performance of orthopedic-related coatings. The aim of this study is the qualitative, systematic review of the in vitro and in vivo properties of antimicrobial-, silver-, iodine-based, and novel technology of released and contact-type orthopedic implant coatings in order to point out the possible use of these materials in clinical settings and the need to validate any promising new tools to be introduced in the shield against infection.

## 2. Materials and Methods

### 2.1. Protocol

The protocol of the present systematic review was registered in the PROSPERO international register of systematic reviews (registration number: CRD42023444527).

### 2.2. Research Strategy

A systematic computer-based literature review search with predefined criteria was performed according to the Preferred Reporting Items for Systematic Reviews and Meta-Analyses (PRISMA) guidelines [16] in the following databases: PubMed (1947 to 10 August 2023) and Web of Science (1900 to 10 August 2023). Research methodology used a combination of the following terms: “coated implant infection” [All Fields] AND “bone” [All Fields] AND “orthopaedics” [All Fields] AND “in vitro and in vivo [All Fields]”, AND “surface modification” [All Fields].

All the electronic literature search was conducted independently by two authors (A.K. and E.P.) and an experienced librarian. Moreover, the above authors independently screened the titles and abstracts to identify relevant studies of outcomes and periprosthetic infection complications after the application of antimicrobial coating. If there was a disagreement between them, the final decision was made by the senior authors (P.J.P. and O.D.S.).

### 2.3. Inclusion Criteria and Study Selection

Studies that examined the outcome of modification in prosthetic surfaces for prophylactic effects against infection in preclinical settings were included in our systematic review. The eligibility criteria were defined according to the acronym PICOS (Population, Intervention, Comparison, Outcome and Study design) such that (P): animals from all species and sexes; (I): application of contact and releasing-type antimicrobial-coated implants; (C): control group without application of coating techniques; (O): studies where the outcome was convincingly and clearly presented; (S): studies that examined the efficacy of the coating techniques in specific micro-organisms compared to the control group. Additional inclusion criteria included (a) studies written in the English language and (b) experimental studies concerning the effectiveness of contact and releasing-type antimicrobial-coated implants in vitro. Contact and releasing-type implant coatings for joint or long-bone applications by any biological or chemical agent were selected. Only full-text articles were eligible for our study. There were no publication date limitations set.

Research that did not include comparative results or was written in a language other than English was excluded. Case reports, reviews, letters to the editor, expert opinions articles, or book chapters with insufficient details about the type of surface modification, the experimental outcome regarding infection rates, osseointegration, biocompatibility, and toxicity effects or studies with non-obtainable data were excluded. Entries with spinal-related implants, referred to as composites, bars, cones, discs, or cylinder plugs, were excluded. All clinical studies were also excluded.

### 2.4. Data Extraction

Two reviewers (A.K. and E.P.) examined all the identified studies and extracted information using a predetermined form. Data from each study were assembled in a Microsoft Excel spreadsheet and classified per orthopedic implant, type of coated prosthesis, cell lines, species of the animal, bacterial strains, and animal model characteristics. The presence of duplicate studies was examined using Endnote 20 software (Clarivate Analytics, Philadelphia, PA, USA).

### 2.5. Quality Assessment

Three reviewers (A.K., E.P., and E.V.) independently evaluated the quality of the included studies. Since different types of studies were included, the 10-scale CAMARADES (The Collaborative Approach to Meta-Analysis and Review of Animal Data from Experimental Studies) [17] and 12-score QUIN [18] quality assessment tools for in vivo and in vitro studies were applied, respectively. CAMARADES and QUIN scores greater than 5 and 12, respectively, were considered of good quality. The CAMARADES SCORE, which is an updated score based on the STAIR SCORE, assesses the quality of animal studies using the following criteria: (a) peer-reviewed publication, (b) statement of control of temperature, (c) random allocation to treatment or control, (d) allocation concealment, (e) blinded evaluation of the published outcome, (f) use of anesthetic without significant alteration of results, (g) appropriate animal model, such as the assessment of the antimicrobial efficacy, osteointegration ability and biocompatibility, (h) sample size calculation, (i) compliance with animal welfare rules and regulations, and (j) statement of potential conflict of interests. The QUIN score is a tool to assess the risk of bias for in vitro studies, which originates from surveys from medical experts who identified key points for a rightly structured study and then verified by other colleagues. The QUIN criteria were (a) clarified aims/objectives, (b) explained sample size calculation, (c) detailed explanation sampling method, (d) details of comparison group, (e) detailed explanation of methodology, (f) operator details, (g) randomization, (h) methods of outcomes measurement, (i) blinding, (j) statistical analysis, and (k) presentation of results.

## 3. Results

### 3.1. Search Results

**Figure 1:**
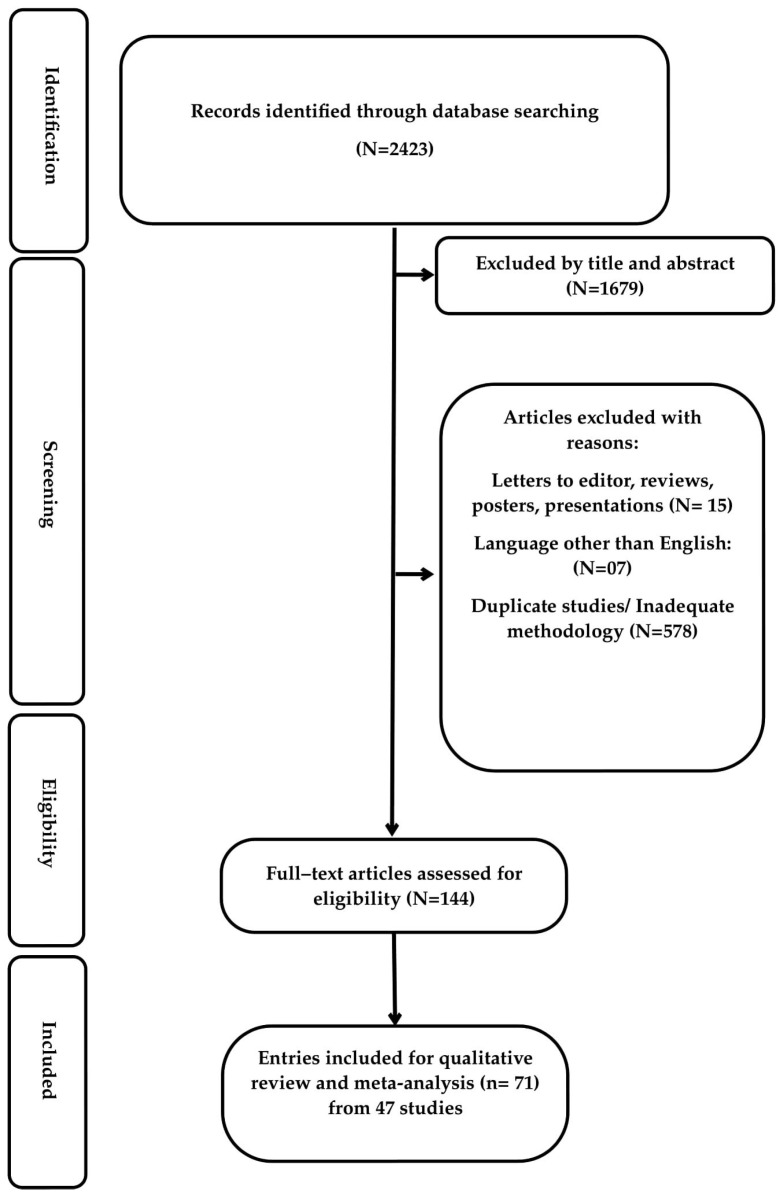
Preferred Reporting Items for Systematic Reviews and Meta-Analysis (PRISMA) flowchart for seeking and identifying included studies.

There were 2423 studies identified from the initial search. After evaluation of the titles and abstracts, we excluded 1679 studies and reviewed the full texts of the remaining 144 studies. Fifteen studies were excluded based on the review of the full text. After reviewing the 129 remaining studies and their bibliographies, 71 entries were included in this systematic review (Figure 1). Entries refer to in vitro and in vivo observations; one study (publication) could have two entries corresponding to in vitro and in vivo results.

### 3.2. Study Design and Content

#### 3.2.1. In Vitro Studies and Cell Lines

The in vitro studies included in this work (31 entries in total) were published between 2005 and 2023. Table 1, Table 2 and Table 3 contain representative coatings/implants, which have been evaluated for their antimicrobial efficacy and biocompatibility in vitro and are suitable for orthopedic applications. Coatings/implants were categorized into four groups, namely antimicrobial-based, silver-based, iodine-based, and novel coatings. Antimicrobial-based coatings were assessed in 12 entries (1 of which was not accompanied by in vivo findings) (Table 1) [7,19,20,21,22,23,24,25,26,27,28,29], silver-based coatings in 10 studies (Table 2) [4,8,11,30,31,32,33,34,35,36], iodine-based in 1 study [37] (Table 2) and under the umbrella of the novel-applied coating technologies (Table 3) were 8 entries (3 of which were not accompanied by in vivo findings) [9,38,39,40,41,42,43,44].

Regarding the 12 studies on antimicrobial-based coatings, the majority were antibiotic-based (synthetic or not). Two studies examined the effects of a synthetic biofilm inhibitor [20,25] and two examined the effect of three antiseptics in total [23,26]. There were three studies where more than one antimicrobials were assessed simultaneously; in Harris et al. (2017), the antibiotic amikacin and the biofilm inhibitor cis-2-decenoic acid (C2DA) were used together [20], in Kalicke et al. (2006), two antibiotics and two antiseptics were examined within the same study [24], and in Kaur et al. (2014), combination therapy consisting of phage and linezolid was reported [22]. In terms of the 10 silver-based coatings, three studies examined silver in nanoparticles (NPs) [4,29,30]. There was one report with in vitro findings for iodine-based coatings [37]. Concerning the eight novel coatings, these involved metals such as copper (Cu) [9,41] and zinc (Zn) [42], non-metals such as selenium (Se) [38], and other agents such as red phosphorus (RP) [39], VE [39], and the AMPeps HHC36 and beta-defensin (MBD-14) [43,44].

#### 3.2.2. In Vivo Studies and Implants

**Figure 2:**
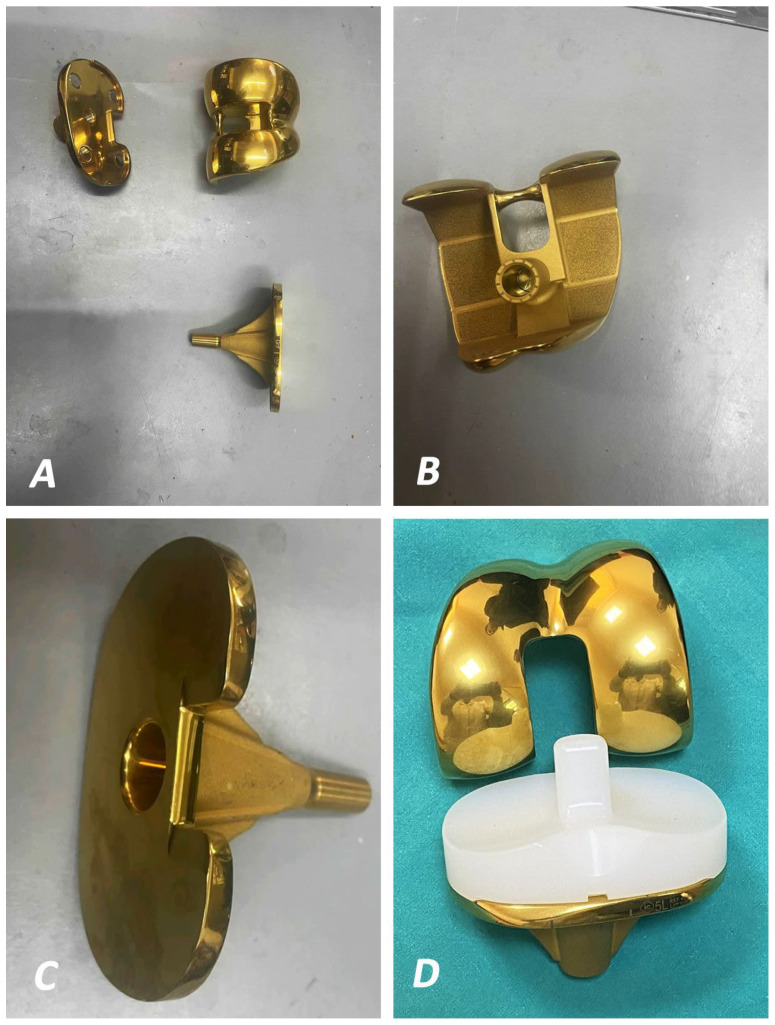
Titanium nitride (TiN)-coated implants for total knee arthroplasty (**A**) composed of the femoral (**B**), tibial (**C**) components, and the polyethylene insert (**D**) displaying significant anti-infective activity and excellent biocompatibility linked to controlled ion release and long-term chemical stability.

**Figure 3:**
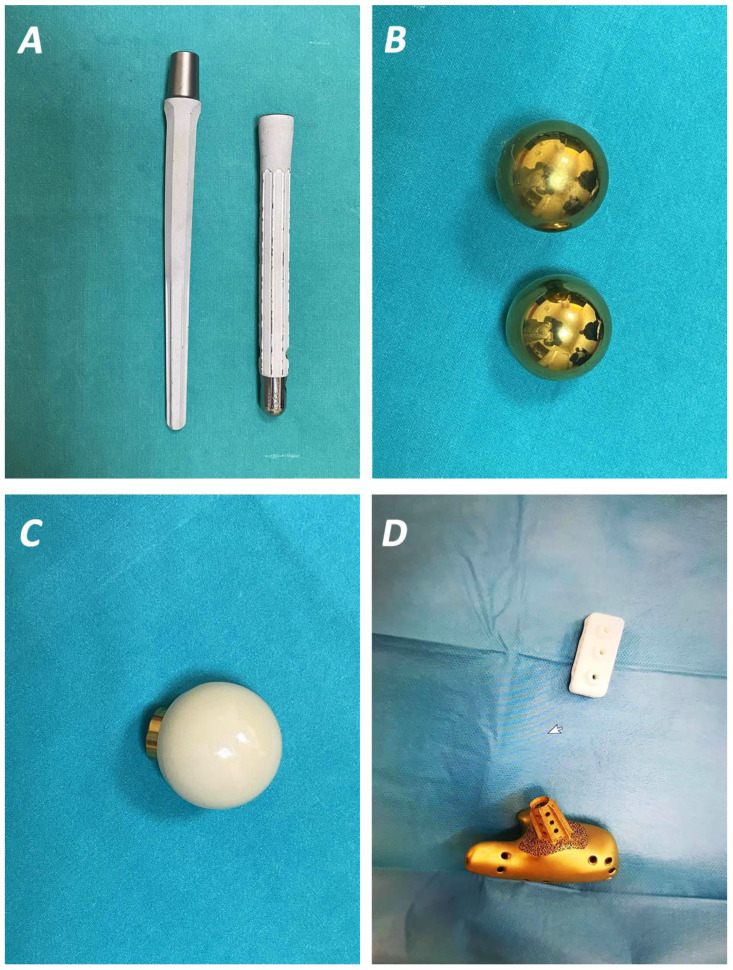
Silver-coated femoral stem (**A**), titanium nitride (TiN) (**B**), and vitamin E-coated (**C**) femoral heads applied in patients after a two-stage revision for infected total hip replacement. (**D**) custom-made titanium nitride (TiN)-coated implant fabricated with 3D printing technique for the replacement of the calcaneus after complex osteomyelitis.

This manuscript has evaluated 40 in vivo entries (24 of them dealing with both in vivo and in vitro observations), which were published from 2009 to 2023. Research data from these studies are presented in Table 4 and Table 5 [4,20,21,22,23,32,33,34,35,36,43,45,46,47,48,49,50,51,52,53,54,55,56,57,58,59,60,61,62,63]. Different animal models have been used, including rats, in most cases (18 studies), while sheep were used as a model in one case [45]. The implants used consisted of a large variety of titanium or stainless steel K-wires, rods, nails, pins for external fixations, alloy bolds, screws, rectangular implants, and hydrogels (Figure 2 and Figure 3). Antimicrobial-based coatings have been evaluated in 23 of these studies (one of them combined the use of vancomycin, VA, with a silver-coated implant) (Table 4) [46,64], while silver-coated implants (Figure 3A) have been used in 12 studies [4,31,32,33,34,35,36,46,55,56,61,62,63]. The use of novel coatings is being studied in the rest of the cases (Table 5) [39,47,48,52].

#### 3.2.3. Bacterial Strains and Antimicrobial Effectiveness

Assessment of the antimicrobial efficacy of the reported coatings was performed against a variety of bacteria (Table 1, Table 2 and Table 3), with more prominence observed for *S. aureus* (in 24 entries), followed by *S. epidermidis* (in eight studies), *P. aeruginosa* (in five studies), *MRSA* (in four studies), *E. coli* (in five studies), and *B. subtilis*, doxycycline (doxy) susceptible *MSSA* and *S. epidermidis* (methicillin-resistant) in one study each. In the majority of studies, one pathogen was used as a means of infection, while there were seven studies where two different bacteria were used and five studies where three or more were used in different assays (Table 1, Table 2 and Table 3). Similarly, with the in vitro cases, the majority of the in vivo ones studied the antimicrobial effects on *S. aureus* (26 of them). Other Gram-positive bacteria under investigation included *MRSA* (eight cases) and *S. epidermidis* (three cases), while Gram-negative bacteria, including *P. aeruginosa* (four cases) and *E. coli* (five cases), were also used. Finally, a combination of two or more bacteria has been reported in 12 studies. Infections during surgery or postoperatively are characterized by bacterial adhesion, subsequent colonization, and, ultimately, the formation of biofilms. Antibacterial efficacy (effect on bacterial growth, adhesion, biofilm formation, and even on the occurrence of resistance) of these coatings was assessed in vitro with standard microbiological assays, such as the colony-counting method, inhibition zone assay, and microscopic techniques. In many cases, antibacterial activity was measured after the release of the agent in question from the coating or following the adhesion of bacteria onto the implant. The antimicrobial activity of the majority of these coatings has also been evaluated in vivo (Table 4 and Table 5); the studies that are accompanied by in vivo observations are marked by an asterisk in Table 1, Table 2 and Table 3.

#### 3.2.4. Osteointegration Ability and Biocompatibility

For implants that are intended for long-term use, besides antimicrobial efficacy, osseointegration ability is highly desired. Interestingly, a well-osseointegrated implant is less susceptible to bacterial infection [11]. To assess biocompatibility, a plethora of relevant bone-related cell lines were used (Table 1, Table 2 and Table 3), with more prominent being the murine osteoblast cell line MC3T3-E1 (in eight studies), the human bone osteosarcoma cell lines MG-63 (in three studies), and Saos-2 (in two studies), as well as other cell lines, including primary osteoblasts, human microvascular endothelial cells (HMVEC), fibroblasts, and mesenchymal stem cells (MSCs). No effect regarding biocompatibility was observed in all in vivo studies, as summarized in Table 1.

Viability and adhesion of human cells are important for osseointegration and bone repair. The effect of coatings on the morphology, viability, and adhesion of cells was assessed (Table 1, Table 2 and Table 3) via proliferation/cytotoxicity assays and microscopic evaluation of the cells. Osteogenic differentiation and osteogenesis were also widely estimated by assessing the activity of alkaline phosphatase (ALP), quantifying osteogenesis-related genes, and using the mineralization assay and deposition of calcium nodules. Among the genes that are central to bone turnover are ALP, a marker for early bone differentiation/maturation; osteocalcin (OC), a marker of late-phase osteogenic differentiation and bone mineralization; collagen-I (Col-1), an abundant component of the extracellular matrix; and runt-related transcription factor 2 (runx2), an early stage osteogenetic transcription factor. In vivo, osseointegration and osteogenesis have been summarized in Table 4 and Table 5.

#### 3.2.5. Quality Assessment

CAMARADES and QUIN assessment tools that were used to evaluate the quality of the included experimental studies demonstrated good quality of the included studies.

## 4. Discussion

Although several strategies, such as aseptic techniques and the use of antibiotics, have predominated in the current prophylaxis of infection in orthopedic interventions, the prevalence of periprosthetic infections in orthopedic surgery remains high. Regarding the promising clinical results of coating techniques in the prevention of implant infections, further research on novel in vitro and in vivo research findings may provide not only an increased understanding of the current applied techniques but also novel therapeutic approaches in biofilm reduction [12]. To the best of our knowledge, this is the first systematic review of released and contact-type coating techniques that analyzed the results of both in vitro and in vivo studies, providing robust evidence about the antimicrobial and osteoinductive activities along with the biocompatibility of these materials.

### 4.1. Evaluation of Antibiotic-Based Coatings

All antibiotic-based coatings, namely gentamicin [7], vancomycin (VA) [8], amikacin [20], doxy [21], linezolid [22], rifampicin (RFP), and fusidic acid [24], showed very good antibacterial activity. This was also the case for antiseptic-based coatings, namely octenidin, irgasan [24], and chlorhexidine (CHX) [23]. Interestingly, when the antibiotics RFP and fusidic acid were compared against the antiseptics octenidin and irgasan, the latter showed more pronounced effects [22]. According to Harris et al. (2017), while 15% amikacin alone had no effect when combined with C2DA, it inhibited bacterial growth [20]. A combination of linezolid and a phage, in Kaur et al. (2014), reduced bacterial adhesion, and inhibition was statistically significant compared to each of the agents alone at every time point examined [22]. Of the 12 studies of antimicrobial-based coatings, 5 examined their biocompatibility in cellular systems [7,19,26,27,28]; there was no effect on cell growth/viability [7,19,26,27,28] nor on osteogenesis-related markers [7,29].

Gentamicin has a broad bactericidal spectrum and appears to be non-toxic and biocompatible [7]. Emerging resistance, however, to gentamicin poses a serious problem [21]. VA is a glycopeptide with a broad antimicrobial spectrum, which extends to methicillin-resistant strains [19]. Doxy is a broad-spectrum antibiotic, and its low resistance (even for *MRSA*) is documented. It is less nephrotoxic and enters host cells more efficiently than gentamicin [20]. Linezolid, a synthetic antibiotic, has a low potential of developing intrinsic resistance and does not show cross-resistance to other systemically administered antibiotics. Linezolid has 100% oral bioavailability, good pharmacokinetics, and good osteo-articular tissue penetration [22,65,66,67,68,69]. The selected MR-5 lytic phage, which is a broad-spectrum bacteriophage, represents a simple, inexpensive, and safe tool. As transduction of virulence or resistance genes is minimal, phages can self-multiply in the tissue surrounding the implants for as long as the bacteria are present without having adverse effects or causing tissue toxicity [8]. The benefits of the combination of phage and linezolid were supported by the biocompatibility of hydroxypropyl methylcellulose (HPMC). Release of the antibiotic amikacin and the biofilm inhibitor C2DA have been suggested to have synergistic effects against a variety of pathogens. The added value of the presence of C2DA is that it lowers the amount of antibiotic that needs to be loaded onto the coating [20]. An envisaged sequence of action would have amikacin act first by killing bacteria, while C2DA would work afterward by delaying/preventing bacterial adhesion and biofilm formation, allowing time for the antibiotics or the immune system to respond [20]. In terms of the phosphatidylcholine (PC) coating, it is envisaged that PC liposomes can be formed following erosion of PC from the coating, which will contain amikacin and C2DA. These liposomes will extend the elution period [20]. RFP and fusidic acid both have broad spectra of effect, including biofilm-producing bacteria. They are complementary to each other in terms of the bactericidal and bacteriostatic actions and together can minimize the risk of occurrence of resistance. They can also penetrate the tissue and exert their effect around the bone and in the surrounding tissue. Octenidin and Irgasan also have broad spectra of action. The antiseptics have a faster outcome compared to the more delayed action of the antibiotics, as they directly attack the bacterial cell membrane, contrary to inhibition of the bacterial DNA-dependent RNA synthesis or inhibition of bacterial RNA polymerase and of protein synthesis, that each of the antibiotics RFP and fusidic acid causes, respectively. In terms of the poly-L-lactide (PLLA) matrix, it ensured mechanical stability, while its gradual degradation was essential for the release of the antimicrobial substances incorporated there [21]. Moxifloxacin, as used in sol-gel coatings, provides anti-infective activity both in vivo and in vitro in Ti implants [27]. This activity summarizes the inhibition of biofilm formation and mature biofilm treatment. Chlorhexidine (CHX), one of the frequently used antiseptics, has a broad spectrum of activity. The inclusion of dopamine increases adhesion to metallic substrates [23]. Finally, the use of fosfomycin seems not to be effective regarding bacterial eradication and the prophylaxis of biofilm formation [57]. However, an important drawback of antimicrobial-based delivery systems is the continuous decrease in the antimicrobial’s concentration. In addition, as the development of bacterial resistance is a complication of antibiotic therapy, different coatings exhibiting antimicrobial properties have been developed and presented in Table 1 and Table 2.

Controlled delivery of antimicrobial-based coatings through transfer systems with high encapsulation ability has already been used to enhance the antimicrobial capacity. Mesoporous silica nanoparticles (MSNs) have been tested in order to encapsulate CHX, and the combination was then incorporated in Polydimethylsiloxane (PDMS), ending up in a thin coating film used to investigate possible medical and dental aspects. The results of the in vitro study revealed higher biocompatibility and antibacterial rate without accompanying toxicity of the combined coating substance [70]. Finally, the results of this in vitro and other similar studies seem to be promising regarding the development of new coating systems combining encapsulation technology to achieve synergistic antibacterial properties [71].

### 4.2. Evaluation of Ag-Based Coatings

All silver (Ag)-based coatings, irrespective of whether they were bare or as nanoparticles (NPs), exhibited antimicrobial activity, with the majority reporting inhibition of bacterial growth, adhesion, and biofilm formation [8,11,30,31,34,35]. All but one study examined biocompatibility and reported a lack of any negative effect on cell morphology and adhesion, as well as viability/proliferation. Cytotoxicity was only observed at 10 mM and >11.36% silver [34] and, in the case of AgNTs/HA, lacking chitosan [23], which will be discussed later. In terms of osseointegration, three studies confirmed the promotion of osteogenic differentiation and osteogenesis [4,31,35]. Ag is widely used because it exerts broad-spectrum antimicrobial activity against Gram-positive and -negative bacteria, including antibiotic-resistant strains, fungi, protozoa, and certain viruses [33]. In fact, the bactericidal properties of Ag are well-established [41]. Ag is inert but ionizes to Ag^+^ in the presence of body fluids. AgNPs also release Ag^+^. Ag^+^ is known to confer effective antibacterial activity in vitro and in vivo without allowing the development of resistance. Of importance is its ability to prevent biofilm formation. The antibacterial mechanism of Ag^+^ consists of structural changes to the bacterial cell wall, increased permeability, damage of the bacteria’s proteins, DNA, and RNA, disruption of metabolism and inhibition of bacteria respiratory chain, and, ultimately, cell death [31,34]. Nanolayer Ag has the advantage of preventing the release of potentially very toxic quantities of silver whilst retaining its antimicrobial activity [30,31]. When AgNPs are combined with polydopamine (PDA), which itself has antimicrobial activity, better antibacterial efficacy is envisaged [4,68,69,72,73]. In addition, PDA biocompatibility and adhesive properties render it a useful coating [31]. Chitosan (CS), a biopolymer with complexing and chelating properties, allows the sustainable release of Ag^+^ from the coating [31]. The absence of CS from AgNTs/HA could be responsible for the reported cytotoxicity [33]. Immobilization of Ag^+^ via IP6 chelation retains antibacterial efficacy [27]. In Svensson et al. (2013), the antibacterial mode of action was not fully elucidated; it did not seem to be dependent on release but rather on the nanostructure of the coating itself [11]. However, according to several in vivo and limited clinical studies [15], the application of Ag-coated implants (Ag^+^) has proven to be well-tolerated without toxicity or related side effects [23,33,41].

### 4.3. Evaluation of Iodine-Based and Other Novel Coatings

The paper on the iodine-based coating examined solely the antibacterial effect of iodine and reported inhibition of adhesion and biofilm formation. Povidone-iodine is a broad-spectrum (including viruses and fungi) antimicrobial agent with a low propensity for developing resistance or causing toxicity [37].

In the diverse category of novel coatings, almost all showed considerable antibacterial activity; reports on Cu [9], Zn [42], VE [40], and RP [39] demonstrated inhibition of adhesion and biofilm formation. Exceptions were reported for the blended VE implants, where intra-species differences were noticed [40], and for TiCuN + BONIT^®^ [9]. Considering biocompatibility, most coatings were shown not to have any effect on cell viability/proliferation (in some cases, it was even found to be increased) [39,43]. Interestingly, moderate compatibility and decreased viability/proliferation were found for TiCuN and TiCuN + BONIT^®^ 9, 4xCu-TiO2 27, NT-Zn3h 30, and HHC36 AMPep for >200 μg/mL [44]. As far as osseointegration was concerned, three studies reported enhanced osteogenic differentiation and osteogenesis [33,34,36]. The antibacterial efficacy and osteoinductive ability of the aforementioned coatings have also been evaluated in vivo (Table 5), complementing and strengthening the in vitro findings and suggesting that these properties are due to and not impaired by the respective coatings. Specifically, reduced bacterial growth, biofilm formation, and inflammation have been noted by several studies, while osseointegration has either been unaffected [5,7,10,60] or enhanced [31,43,46,53,62]. Moreover, satisfying or excellent biocompatibility has been reported, too [28,29,39].

Selenium NPs damage the bacterial membrane of *MRSA*, thus inducing rapid cell lysis. They are stable due to their inorganic nature and can easily be immobilized on implant surfaces whilst retaining their activity [38]. The advantages of the RP-IR780-RGDC titanium implant are as follows: RP and its degradation products are non-toxic, the small amount of singlet O2 seems to enhance the susceptibility of the bacteria to heat, increase the bacterial membrane’s permeability, and eliminate biofilm, following irradiation with an 808 nm laser. In addition, RGDC seems to improve adhesion and proliferation [39]. VE and its antioxidant properties may be a key point affecting bacterial adhesive ability and biofilm formation. Modifications of the properties of UHMWPE by VE showcased a reduction in adherence of some bacterial strains, with the intraspecies differences, however, suggesting the need for more research in order to fully appreciate the added advantage of VE [40]. A more recent in vivo study showcased that VE phosphate could enhance bone stimulation and deposition [48]. There is increasing interest in determining the antimicrobial and osseointegrative properties of VE as a coating for orthopedic or dental implants. Heavy metal ions, such as Cu ions, can become toxic. Cu can have a bacteriolytic effect and stop bacteria from replicating [9]. The low release of Cu observed for TiCuN + BONIT^®^ could be responsible for the lack of antibacterial properties reported [9]. Cu seems to be effective on planktonic bacteria and bacteria formatting a biofilm while presenting low toxicity. This activity is based on the inhibition of biofilm formation by influencing the advantage of the osteoblasts on the implants’ surface [9]. The higher affinity of AMPs for bacterial membranes renders them suitable for antimicrobial agents with low toxicity peptides to form electrostatic interactions with anionic phospholipid groups of the bacterial membrane, to then disrupt the membrane and cause bacteria death [43,44]. The higher affinity of AMPeps for bacterial membranes renders them suitable for antimicrobial agents with low toxicity [73]. Covalent immobilization of MBD-14 on SP is believed to prevent its rapid degradation and ensure its stability. This association in combination with the porous matrix, might be responsible for the antibacterial activity observed [44]. Similarly, the antimicrobial effect of zinc (Zn) is mainly expressed by Zn complexes and ZnO NPs [74]. Zinc complexes express antifungal activity, whereas ZnO NPs are characterized by antimicrobial activity by two different mechanisms. These activities summarize in the release of reactive oxygen species (photocatalytic process) or ZnO nanoparticles, which lead to the production of intracellular ROS, inducing damage to the cells.

One of the most important limitations of all the nanoparticles used is the lack of available data from in vivo studies with long-term results summarizing the use of these types of implants in animal models. The use of a variety of implants in different animal models provides heterogeneous results, which need to be further specified in future studies.

The presented data of the in vitro and in vivo results of the included studies strongly suggest the application of conventional and novel antimicrobial surface modifications of the implants by orthopedic physicians in the management of postoperative implant infections. However, our systematic review has several limitations. Although 73 entries of high quality were included in this review, the studies’ designs and methods were heterogeneous as different animal models were used and no standardized methods were applied in order to evaluate the reproducibility of the outcomes. Additionally, there are some novel coating techniques that have not been tested in vivo. The lack of experience in clinical settings raises concerns about the long-term results of these implants and the growth of multidrug-resistant micro-organisms as a result of their clinical use. Finally, a language bias could be present as only studies written in English were reviewed.

## 5. Conclusions

Assessment of both in vivo and in vitro studies revealed a wide antimicrobial spectrum of the coated implants under investigation, related to inhibition of biofilm formation and unaffected or enhanced osteointegration, as expressed through the impact of various cells on surface attachment or proliferation. Moreover, the use of these implants was often not related to elevated toxicity levels. Taking into account the known limitations associated with the use of different types of coated implants, their presence can be regarded as a promising candidate for the efficient treatment of implant-related infections. Results from in vitro studies involving both novel coatings and the use of encapsulation technology could aid in the design of effective antibacterial coating materials with high biocompatibility and nontoxicity. Finally, these outcomes should be further studied and validated through clinical trials to be used in clinical practice in the future.

## Figures and Tables

**Table 1:** In vitro studies with antimicrobial-based coatings.

Author Year	Coating Technology and Type of Implant ^a^	Release Profile Burst Release	Antimicrobial Activity		
			Pathogens	Outcome	Cells
Vester et al. [7], 2010 *	Gentamicin (10% *w*/*w*) PDLLA (10% *w*/*w*) Ti IMnails and K-wires	Yes (60% within 1 min, 85% after 6 w)	*B. subtilis*, *S. aureus*, *S. epidermidis*	Bactericidal effect, adhesion inhibition, no development of resistance	Saos-2
Zhang et al. [19], 2014 *	VA-coated Ti implants	Yes (~50% on d1, ~80% through d28)	*S. aureus*	Growth inhibition	MC3T3-E1
Harris et al. [20], 2017 *	Amikacin and C2DA (5–25% w/w) PC-coated stainless steel K-wires	Yes (mainly for 1–2 d, 40–50% through d4-7)	*P. aeruginosa*, *S. aureus*	Growth inhibition (*S. aureus*: 25% amikacin or 15% amikacin + C2DA, *P. aeruginosa*: all tested eluates; no inhibition of *S. aureus* for 15% amikacin alone or 5% amikacin + C2DA)	-
Metsemakers et al. [21], 2015 *	Doxy-loaded PLEX-coated TAN rectangular implants or IM nails	Yes (25% on d1, >95% through 4 w)	doxy^R^ *MRSA*, doxy^S^ *MSSA*	Growth inhibition	-
Kaur et al. [22], 2014 **	Phage and Linezolid (5% w/w) HPMC (4% w/v)-coated K-wires	Max. elution (linezolid, within 30 min; phage, after d1, both through d4)	*MRSA*	Adhesion inhibition, no development of resistance	-
Riool et al. [23], 2017 *	CHX (5, 10 wt %)/dopamine/epoxy-based Al sheets and Ti implants	Yes (>80% within d1, through d4)	*S. aureus*	Bactericidal effect	-
Kalicke et al. [24], 2006 *	RFP (3%) and fusidic acid (7%) or Octenidin (2%) and Irgasan (8%) PLLA-coated Ti plates	Yes (~60% within 1 h, ~80% after 42 d)	*S. aureus*	Bactericidal effect, adhesion inhibition (more pronounced in the antiseptic-coated plate)	-
Miao, et al. [25], 2021 *	HHC36-PDLLA/PLGA implants	Yes (present in the first hours, 30% for the PDLLA group and 21% for the PLGA group on d1, 47, and 33%, respectively, after 15 d)	*S. aureus*	Bactericidal effect, adhesion inhibition	-
Yu, et al. [26], 2021 *	(MMT/PLL-VA)_8_ K-wires	Yes (CMS degradation accelerates multilayer degradation and VA release)	*S. aureus*	Bactericidal effect	Osteoblasts
Aguilera-Correra, et al. [27], 2019 *	Moxifloxacin-loaded organic–inorganic sol-gel Ti K-wires	Yes (linear release with max rate at 48 h)	*E. coli*, *S. aureus*, *S. epidermidis*	Biofilm formation inhibition	MC3T3-E1
Bai, et al. [28], 2013 *	(MMT/HA-RFP)_10_ K-wires (RFP: 1 mg/mL)	-	*S. aureus*	Growth inhibition	-
Liao, et al. [29], 2021 *	(MMT/PLL-CHX)_10_ K-wires	Slow CHX release in PBS, increased release in the presence of *S. aureus*	*S. aureus* (CMS)	Bactericidal effect	Osteoblasts

* The studies which are accompanied by both in vitro and in vivo assessments of the coatings are marked by an asterisk. ** This study was not accompanied by in vivo observations but is nonetheless presented as it provides insight into the coating technology/implant in question. ^a^ Under “coatings/implants”, implants used both in in vitro and in vivo assays are reported. Al, aluminum; ALP, alkaline phosphatase; *B. subtilis*, *Bacillus subtilis*; C2DA, cis-2-decenoic acid; CHX, chlorhexidine; CICP, C-terminal propeptide of type I collagen; CMS, chymothrypsin; d, day; doxy^R^, doxycycline-resistant; doxy^S^, doxycycline susceptible; *E. coli*; *Escherichia coli*; HA, hyaluronic acid; HHC36, KRWWKWWRR; HPMC, hydroxypropylmethlycellulose; IM, intramedullary; K-wires, Kirschner-wires; MMT, Montmorillonite; *MRSA*, methicillin-resistant *Staphylococcus aureus*; *MSSA*, methicillin-susceptible *S. aureus*; *P. aeruginosa*, *Pseudomonas aeruginosa*; PC, phosphatidylcholine; PDLLA, poly-D,L-lactide; PLEX, polymer–lipid encapsulation matrix; PLGA, poly lactic-co-glycolic acid; PLL, poly-L-lysine; PLLA, poly-L-lactide; RFP, Rifampicin; *S. aureus*, *Staphylococcus aureus*; *S. epidermidis*, *Staphylococcus epidermidis*; TAN, titanium aluminum niobium; Ti, titanium; VA, Vancomycin; w, week.

**Table 2:** In vitro studies with silver and iodine-based coatings.

Author Year	Coating Technology and Type of Implant ^a^	Release Profile Burst Release	Antimicrobial Activity		Biocompatibility	
			Pathogens	Outcome	Cells	Outcome
Xu et al. [4], 2018 *	AgNPs/PDA-coated PEGda hydrogel	-	*E. coli*, *S. aureus*	Bacteriostatic effect (more pronounced in *E. coli*)	MC3T3-E1	No effect on morphology and adhesion, better viability, promotion of osteogenic differentiation and osteogenesis (increase in ALP, BSP, OC, and Runx2 mRNA expression, increased mineralization)
Honda et al. [8], 2013 *	Ag (1–20 mol%)/HAp powders	Yes (high within 2 d)	*S. aureus*	Bactericidal effect, adhesion and biofilm formation (>5 mol%) inhibition	MC3T3-E1	No effect on viability (only 5 mol% was tested)
Svensson et al. [11], 2013 *	Ag/Pd/Au-coated Ti screws	-	*S. aureus*	Adhesion inhibition	-	-
Devlin-Mullin et al. [30], 2017 *	AgNPs-coated Ti solid and foam implants	-	*MRSA*, *S. epidermidis*	Adhesion and biofilm formation inhibition (on *S. epidermidis*, no effect on *MRSA*)	Saos-2, HMVEC	No effect on morphology, viability and adhesion
Xie et al. [31], 2019 *	AgNPs/HAp/CS/PDA-coated Ti nails	-	*E. coli*, *S. aureus*, *S. epidermidis*	Adhesion and biofilm formation inhibition, regulation of biofilm-related genes (icaA, icaR)	MC3T3-E1	No effect on viability (but cytotoxicity on AgNTs/HAp), enhanced osteogenic differentiation (increased ALP activity and mineralization)
Shevtsov et al. [32], 2019 *	Ag-coated Ti tablets and SBIP	-	*P. aeruginosa*, *S. aureus*, *S. epidermidis*	Adhesion and biofilm formation inhibition (including of planktonic bacteria)	MG-63, dermal fibroblasts, MSCs	No effect on morphology and adhesion
Funao et al. [33], 2016 *	Ag^+^ (0.1–10)/HAp/IP_6_ Ti pins	Yes (plateau by d1 + d3 depending on [Ag^+^], through d7)	*S. aureus*	Growth inhibition (1–10 mMAg^+^)	L-929 fibroblasts	No effect on viability (<20% at 5 mM Ag^+^, >50% at 10 mM)
Tran et al. [34], 2013 *	Ag (1.8–11.36 wt%)/TiO/siloxane-coated stainless steel IM nails	-	*S. aureus*	Bactericidal effect, adhesion inhibition (>1.8%)	Osteoblasts	No effect on viability (cytotoxicity for >11.36%)
Kuo et al. [35], 2022 *	SrMBG (10 wt% Sr) and AgSrMBG (10 wt% Sr and 1.64 wt%) Ag powders/ PEM films	Yes (at d8, PEM >57% of weight lost, PEM/ SrMBG 43%, PEM/AgSrMBG 37%)	*E. coli*	Growth inhibition	-	-
Hu et al. [36], 2020 *	TaN-Ag, TaN-(Ag, Cu), TaON-Ag, and TaN-coated Ti needles	-	*CoNS*, *E. coli*, *MRSA*, *MSSA*, *P. aeruginosa*	Growth inhibition (of TaON-Ag coating)	MSCs	No effect on osteogenesis
Inoue et al. [37], 2017 *	Iodine-coated (on oxidation film) Ti6Al4V metallic washers and K-wires	-	*S. aureus*	Adhesion and biofilm formation inhibition	-	-

* The studies which are accompanied by both in vitro and in vivo assessments of the coatings are marked by an asterisk. ^a^ Under “coatings/implants”, implants used both in in vitro and in vivo assays are reported. Ag, silver; ALP, alkaline phosphatase; Au, gold; BSP, bone sialoprotein; CS, chitosan; CoNS, coagulase-negative Staphylococcus; Cu, copper; d, day; *E. coli*; *Escherichia coli*; HAp, hydroxyapatite; HMVEC, human microvascular endothelial cells; IM, intramedullary; IP_6_, inositol hexaphosphate; K-wires, Kirschner-wires; *MRSA*, methicillin-resistant *Staphylococcus aureus*; MSCs, mesenchymal stem cells; *MSSA*, methicillin-susceptible *S. aureus*; NPs, nanoparticles; OC, osteocalcin; *P. aeruginosa*, *Pseudomonas aeruginosa*; Pd, palladium; PDA, polydopamine; PEGda, poly (ethylene glycol) diacrylate; PEM, polyelectrolyte multilayer; Runx2, runt-related transcription factor 2; *S. aureus*, *Staphylococcus aureus*; SBIP, skin, and bone integrated pylons; *S. epidermidis*, *Staphylococcus epidermidis*; TAN, titanium aluminum niobium; Ti, titanium; Ti6Al4V, titanium alloy.

**Table 3:** In vitro studies with novel coating techniques.

Author Year	Coating Technology and Type of Implant ^a^	Release Profile Burst Release	Antimicrobial Activity		Biocompatibility	
			Pathogens	Outcome	Cells	Outcome
Bergemann et al. [9], 2017 **	TiCuN and TiCuN + BONIT^®^ films	Yes (high within 24 h for TiCuN, low for TiCuN + BONIT^®^)	*S. epidermidis*	Biofilm formation inhibition (including planktonic bacteria) for TiCuN	MG-63	Reduction in initial adhesion (for TiCuN; enhanced for TiCuN + BONIT^®^), no effect on morphology (less spreading on TiCuN + BONIT^®)^, inhibition of viability (for both implants and with 2 different culturing approaches)
Tran et al. [38], 2019 *	Se (0.25–128 ppm) NPs on Ti plates and screws	-	*MRSA*, *S. epidermidis*	Growth inhibition (as low as 0.5 ppm Se; for >32 ppm, no difference)	hOBs	No effect on morphology, viability, and adhesion
Tan et al. [39], 2018 *	RP–IR780–RGDC Ti implants and rods	-	*S. aureus*	Growth and biofilm formation inhibition (upon irradiation and at 50 °C)	MC3T3-E1	Improved viability, adhesion, and promotion of osteogenic differentiation (increased ALP activity and ALP, OC, and Runx2 mRNA expression)
Gomez-Barrena et al. [40], 2011 **	VE (0.4, 3 wt% doped) or (0.1% blended) UHMWPE disks and squares, respectively	-	*S. aureus*, *S. epidermidis*	Adhesion inhibition (of *S. epidermidis* for both 0.4 and 3%, intra-species differences for 0.1% blended (inhibition of a collection strain of *S.aureus*, but not of clinical strains, while inhibition of 2 clinical strains of *S.epidermidis*, but not of the collection strain)]	-	-
Heidenau et al. [41], 2005 **	Cu-TiO_2_ and 4xCu-TiO_2_-coated Ti6Al4V round metal plates	-	*S. aureus*	Adhesion inhibition (slight for Cu-TiO_2_; pronounced for 4xCu-TiO_2_, including of planktonic bacteria for 4xCu-TiO_2_)	MC3T3-E1	No effect on viability (for Cu-TiO_2_ compared to TiO_2_; increased compared to Ti6Al4V), decreased viability (for 4xCu-TiO_2_), slight effect on morphology (“injured”, dead cells)
Li et al. [42], 2014 *	Zn/TiO_2_-NTs-coated Ti substrates	Yes (max. during d1, through d30 especially for NT-Zn3h)	*S. aureus*	Adhesion inhibition (including of planktonic bacteria; more pronounced for NT-Zn3h)	MC3T3-E1	No effect on morphology (improved spreading), no effect on viability (decreased for NT-Zn3h on d4), no effect on initial adhesion, promotion of osteogenic differentiation (increased ALP activity, ALP, Col-1, OC, and OPG mRNA expression, and matrix mineralization)
Yuan et al. [43], 2019 *	MBD-14 (2, 5, 10 μg/mL)-loaded PEEK (SP) rectangular and cylindrical samples	-	*P. aeruginosa*, *S. aureus*	Growth inhibition (especially for 5, 10 μg/mL)	MSCs	Enhanced viability, adhesion, and osteogenic differentiation (increased ALP activity, increased ALP, Col-1, and OC mRNA and protein expression)
Kazemzadeh-Narbat et al. [44], 2012 *	HHC36 AMPep-loaded CaP-coated Ti plates and cylindrical implants	Yes (approx. 70% within 30 min, 90% within d1, through d7)	*P. aeruginosa*, *S. aureus*	Bactericidal effect	MG-63	No effect on viability (cytotoxicity observed for >200 μg/mL), increased adhesion

* The studies which are accompanied by both in vitro and in vivo assessments of the coatings are marked by an asterisk. ** These studies were not accompanied by in vivo observations but are nonetheless presented as they provide insight into the coating technology/implant in question. ^a^ Under “coatings/implants”, implants used both in in vitro and in vivo assays are reported. ALP, alkaline phosphatase; AMPep, antimicrobial peptide; CaP, calcium phosphate; Col-1, collagen-I; Cu, copper; Cu-TiO_2_, Cu-containing sol-gel-derived; d, day; HHC36, KRWWKWWRR; hOBs, human osteoprogenitor cells; MBD-14, mouse beta-defensin-14; *MRSA*, methicillin-resistant *Staphylococcus aureus*; NPs, nanoparticles; NTs, nanotubes; NT-Zn1h and 3h, samples fabricated by hydrothermal process for 1 h and 3 h, respectively; OC, osteocalcin; OPG, osteoprotegerin; *P. aeruginosa*, *Pseudomonas aeruginosa*; PEEK, polyetheretherketone; RGDC, arginine-glycine-aspartic acid-cysteine; RP, red phosphorus; Runx2, runt-related transcription factor 2; *S. aureus*, *Staphylococcus aureus*; *S. epidermidis*, *Staphylococcus epidermidis*; Se, selenium; SP, sulfonated PEEK group + hydrothermally treated; Ti, titanium; Ti6Al4V, titanium alloy; TiCuN, titanium-copper-nitride; TiO_2_, titanium dioxide; UHMWPE, ultra-high molecular weight polyethylene; VE, vitamin E; Zn, zinc.

**Table 4:** In vivo research data of antibiotic-coated internal fixation and prostheses implants.

Author Year	Animal Model	Coating Technology and Type of Implant ^a^	Antimicrobial Activity and Biocompatibility	
			Pathogens	Outcomes
Stavrakis et al., 2016 [1] *	Mice	VA and Tigecyclin PEG-PPS Ti K-wires	*S. aureus*	Reduction in bacterial forming colonies and of infection osteolysis
Kucharikova et al., 2016 [5] *	Mice	VA and Caspofungin in 3 aminopropyl-triethoxy silane Ti round disks	*C. albicans*, *S. aureus*	Biofilm formation reduction, no effect on osseointegration
Vester et al., 2010 [7] *	Rats	Gentamicin PDLLA Ti IM nails and K-wires	*S. aureus*, *S. epidermidis*	Prevention of bacterial adhesion and resistance, no effect on osseointegration
Gerits et al., 2016 [10] *	Mice	SPI031 Ti disks	*P. aeruginosa*, *S. aureus*	Growth and adhesion inhibition, no effect on osseointegration
Harris et al., 2017 [20] *	Mice	Amikacin and C2DA PC-coated stainless steel K-wires	*P. aeruginosa*, *S. aureus*	Biofilm formation reduction
Metsemakers et al., 2015 [21] *	Mice, Rabbits	Doxy-loaded PLEX-coated TAN rectangular implants or IM nails	*MRSA*, *MSSA*	Complete protection and infection reduction against implant-associated *MSSA* and *MRSA* osteomyelitis, respectively
Kaur et al., 2016 [22] ^#^	Mice	Phage and Linezolid HPMC-coated Ti K-wires	*S. aureus*	Reduced bacterial adherence and inflammation and faster resumption of limb motor function
Riool et al. 2017 [23] *	Mice	CHX/ dopamine/epoxy-based Al sheets and Ti implants	*S. aureus*	Bactericidal effect, reduction in colony forming units, well-tolerated with no-toxicity
Yu et al., 2021 [26] *	Rats	(MMT/PLL-VA)_8_ Ti K-wires	*S. aureus*	Bactericidal effect
Aguilera-Correa et al., 2019 [27] *	Mice	Moxifloxacin-loaded organic–inorganic sol-gel K-wires	*E. coli*, *S. aureus*	Prevention of prosthetic joint infection
Bai et al., 2023 [28] *	Rats	(MMT/HA-RFP)_10_ Ti K-wires	*S. aureus*	Analysis of biofilm formation revealed antibacterial activity, good biocompatibility
Liao et al., 2021 [29] *	Rats	(MMT/PLL-CHX)_10_ Ti K-wires	*S. aureus*	Antibacterial activity, good biocompatibility
Yuan et al., 2019 [43] *	Rats	MBD-14-loaded PEEK (SP) rectangular and cylindrical samples	*P. aeruginosa*, *S. aureus*	Antibacterial activity, good osseointegration
Williams et al., 2019 [45] ^#^	Sheep	CZ-01127 compound on silicone polymer Ti cylindrical plugs	*MRSA*	Local bacteria eradication of normal bone ingrowth
Peeters et al., 2019 [49] *	Rats	5-aryl-2-aminoimidazole compound covalently attached to open porous Ti implants	*S. aureus*	Biofilm formation reduction, no effect on osseointegration
Shiels et al., 2018 [50] *	Rats	CHX polymer layer Ti K-wires	*N/A (contaminated wound)*	Reduced bacteria colonization and osteolysis, increased fracture union
Liu et al., 2017 [51] ^#^	Rabbits	NTATi-G	*S. aureus*	Bacterial growth inhibition, increased bone volume
Song et al., 2013 [53] ^#^	Rats	Doxy coaxial PCL/PVA electrospinning nanofiber Ti pins	*S. aureus*	Bacterial growth inhibition, enhanced osseointegration
Jennings et al., 2016 [54] ^#^	Rabbits	VA-loaded PC Ti wires	*S. aureus*	Reduction in colony forming units, infiltration of inflammatory cells, increased bone growth
Gulcu et al., 2016 [57] ^#^	Rats	Gentamicin and Fosfomycin PDLLA stainless steel K-wires	*S. aureus*	Fosfomycin is not effective in bacterial prophylaxis
Alt et al., 2014 [59] ^#^	Rabbits	RFP-fosfomycin-coated Ti K-wires	*MRSA*, *MSSA*	Reduction in infection susceptibility
Giavaresi et al., 2014 [60] ^#^	Rabbits	VA-loaded DAC Ti sand-blasted IM nails	*MRSA*	Reduction in bacterial colonization, increased histocompatibility
Moojen et al., 2009 [64] ^#^	Rabbits	Tobramycin perapatite Ti cylindrical implants	*S. aureus*	Reduction in infection susceptibility increased osseointegration

* The studies which are accompanied by both in vitro and in vivo assessments of the coatings are marked by an asterisk. ^#^ These studies reported only in vivo observations. ^a^ Under “coatings/implants”, implants used both in in vitro and in vivo assays are reported. Al, aluminum; C2DA, cis-2-decenoic acid; C. albicans, Candida albicans; CHX, chlorhexidine; DAC, resorbable, antibacterial-loaded hydrogel coating; doxy, doxycycline; *E. coli*; *Escherichia coli*; HA, hyaluronic acid; HPMC, hydroxypropylmethlycellulose; IM, intramedullary; K-wires, Kirschner-wires; MBD-14, mouse beta-defensin-14; MMT, Montmorillonite; *MRSA*: Methicillin-resistant *Staphylococcus aureus*, *MSSA*: Methicillin-susceptible *Staphylococcus aureus*, NTATi-G, nanotubular anodized titanium coated with gentamicin; *P. aeruginosa*, *Pseudomonas aeruginosa*; PC, phosphatidylcholine; PCL/PVA: polycaprolactone/polyvinyl alcohol; PDLLA, poly-D,L-lactide; PEEK, polyetheretherketone; PEG-PPS: poly(ethylene glycol)-poly(propylene sulfide), PLEX, polymer–lipid encapsulation matrix; PLL, poly-L-lysine; RFP, Rifampicin; *S. aureus*, *Staphylococcus aureus*; *S. epidermidis*, *Staphylococcus epidermidis*; SP, sulfonated PEEK group + hydrothermally treated; SPI031: N-alkylated 3, 6-dihalogenocarbazol 1-(sec-butylamino)-3-(3, 6-dichloro-9H-carbazol-9-yl) propan-2-ol, TAN, titanium aluminum niobium; Ti, titanium; VA, vancomycin.

**Table 5:** In vivo research data of internal fixation and prostheses implants coated with silver and novel modifications.

Author Year	Animal Model	Coating Technology and Type of Implant ^a^	Antimicrobial Activity and Biocompatibility	
			Pathogens	Outcome
Xu et al., 2018 [4] *	Rats	AgNPs/PDA-coated PEGda hydrogel	*E. coli*, *S. aureus*	Bacteriostatic activity, maxillary bone defects healing
Xie et al., 2019 [31] *	Rats	AgNPs/HAp/CS/PDA-coated Ti nails	*E. coli*, *S. aureus*, *S. epidermidis*	Bacterial adhesion and biofilm formation inhibition, enhanced osseointegration
Shevtsov et al., 2019 [32] *	Rabbits	Ag-coated Ti tablets and SBIP	*P. aeruginosa*, *S. aureus*, *S. epidermidis*	Biofilm formation reduction, good biocompatibility, no toxicity
Funao et al., 2016 [33] *	Mice	Ag^+^/HAp/IP_6_ Ti pins	*S. aureus*	Antimicrobial activity, reduced osteomyelitis markers, no toxicity
Tran et al., 2013 [34] *	Caprine	Ag^+^/ TiO/siloxane-coated stainless steel IM nails	*S. aureus*	Bacterial adhesion reduction, no effect on osteoblast function, reduced osteolysis and infection serum markers
Kuo et al., 2022 [35] *	Rats	SrMBG and AgSrMBG powders/ PEM films	*E. coli*	Long-term antibacterial, angiogenic, and osseointegration activities
Hu et al., 2020 [36] *	Rats	TaN-Ag, TaN-(Ag, Cu), TaON-Ag, and TaN-coated Ti needles	*E. coli*, *MSSA*	Antibacterial activity, no effect on osseointegration
Tan et al., 2018 [39] *	Rats	RP–IR780–RGDC Ti implants and rods	*S. aureus*	Antibacterial activity, biofilm formation inhibition, excellent biocompatibility
Croes et al., 2018 [46] *	Rats	Ag and VA CS-based Ti rods	*S. aureus*	Reduction in infection rate (by VA, not Ag), increased inflammation and osteoclast formation (by Ag)
Martin et al., 2018 [47] ^#^	Rabbits	Carboxymethyl CS-Zn stainless steel pins	*S. aureus*	Prevention of pin-tract infections
Lovati et al., 2018 [48] ^#^	Rats	VE phosphate Ti K-wires	*S. aureus*	Increased bone deposition
Mauerer et al., 2017 [52] ^#^	Rabbits	4x Cu-TiO_2_ Ti6Al4V bolts	*MRSA*	Reduction in infection rate and blood infection indices
Kose et al., 2016 [55] ^#^	Rabbits	Ag doped HAp Ti nails	*MRSA*	Bacterial growth reduction, no toxicity on osteoblastic function
Tsukamoto et al., 2014 [61] ^#^	Rats	Ag HAp Ti rods	*N/A*	No acute or subacute toxicity
Cheng et al., 2014 [62] *	Rats	Ag-TiO_2_-NT rods	*MRSA*	Increased antibacterial activity and bio-integration properties
Akiyama et al., 2013 [63] ^#^	Rats	Ag-HAp Ti rods	*MRSA*	Increased antibacterial activity and infection rates

* The studies which are accompanied by both in vitro and in vivo assessments of the coatings are marked by an asterisk. ^#^ These studies reported only in vivo observations. ^a^ Under “coatings/implants”, implants used both in in vitro and in vivo assays are reported. Ag, silver; CS, chitosan; *E. coli*; *Escherichia coli*; HAp, hydroxyapatite; K-wires, Kirschner-wires; *MRSA*, Methicillin-resistant *Staphylococcus aureus*; *MSSA*, Methicillin-susceptible *Staphylococcus aureus*; NPs, nanoparticles; NTs, nanotubes; *P. aeruginosa*, *Pseudomonas aeruginosa*; PDA, polydopamine; PEGda, poly (ethylene glycol) diacrylate; PEM, polyelectrolyte multilayer; RGDC, arginine-glycine-aspartic acid-cysteine; RP, red phosphorus; *S. aureus*, *Staphylococcus aureus*; SBIP, skin and bone integrated pylons; *S. epidermidis*, *Staphylococcus epidermidis*; Ti, titanium; VA, vancomycin; VE, vitamin E; Zn, zinc.

## Data Availability

Data is contained within the article.
